# High-Frequency Oscillation vs Mechanical Ventilation for Neonatal Acute Respiratory Distress Syndrome: A Randomized Clinical Trial

**DOI:** 10.1001/jamanetworkopen.2026.0268

**Published:** 2026-03-09

**Authors:** Jie Li, Kaizhen Liu, Qian Yang, Qin Tan, Zhichun Feng, Yuan Shi, Long Chen, Ling-Jun Li

**Affiliations:** 1 Department of Obstetrics and Gynecology, The First Affiliated Hospital of Chongqing Medical University, Chongqing, China; 2 Department of Neonatology, Children’s Hospital of Chongqing Medical University, Ministry of Education Key Laboratory of Child Development and Disorders, Chongqing, China; 3 Bia-Echo Asia Centre for Reproductive Longevity and Equality, Yong Loo Lin School of Medicine, National University of Singapore, Singapore; 4 Department of Neonatology, Women and Children’s Hospital of Chongqing Medical University, Chongqing Health Center for Women and Children, Chongqing, China; 5 Faculty of Pediatrics, PLA General Hospital, Beijing, China; 6 Department of Obstetrics and Gynecology, Yong Loo Lin School of Medicine, National University of Singapore, Singapore; 7 Department of Obstetrics and Gynecology, Li Ka Shing Faculty of Medicine, The University of Hong Kong, Hong Kong; 8 Institute for Human Development and Potential, Agency for Science, Technology, and Research, Singapore; 9 Chongqing Health Center for Women and Children, Chongqing, China; 10 National Clinical Research Center for Child Health and Disorders, Chongqing, China; 11 China International Science and Technology Cooperation Base of Child Development and Critical Disorders, Chongqing, China; 12 Chongqing Key Laboratory of Pediatrics, Chongqing, China

## Abstract

**Importance:**

Key clinical features of neonatal acute respiratory distress syndrome (NARDS) are broadly comparable to those observed in pediatric and adult ARDS; however, evidence is insufficient to recommend high-frequency oscillatory ventilation (HFOV) or conventional mechanical ventilation (CMV) as the preferred first-line therapy.

**Objective:**

To evaluate whether HFOV is superior to CMV in reducing bronchopulmonary dysplasia (BPD) and other neonatal adverse outcomes, including death, among preterm infants (≤34 weeks’ gestational age) with NARDS.

**Design, Setting, and Participants:**

This single-center randomized clinical trial conducted from August 1, 2019, to December 31, 2023, enrolled preterm infants born between 25 weeks 0 days and 34 weeks 6 days of gestation with NARDS who were stabilized with CMV. Data were analyzed from October to December 2024.

**Intervention:**

Participants were randomly assigned to continue CMV or transition to elective HFOV.

**Main Outcomes and Measures:**

The primary outcome was BPD, assessed using 2 definitions: definition 1, that of the 2001 Eunice Kennedy Shriver National Institute of Child Health and Human Development (NICHD) and definition 2, one based on 2019 research. Secondary outcomes included death, retinopathy of prematurity (higher than stage 2), necrotizing enterocolitis (stage 2 or higher), intraventricular hemorrhage (grade 3 or higher), air leak, and hemodynamically significant patent ductus arteriosus. Modified Poisson regression, and Cox proportional hazards regression were applied for outcome risk assessment where applicable.

**Results:**

A total of 386 preterm infants (230 male [59.6%]; mean [SD] maternal age, 29.9 [4.8] years) were randomized: 181 to elective HFOV and 205 to CMV. Overall, 154 (39.9%) and 83 (21.5%) developed BPD according definitions 1 and 2, respectively. Elective HFOV reduced the risk of BPD by 24.0% (34.3% vs 44.9%; relative risk, 0.76; 95% CI, 0.59-0.98) according to definition 1 and by 32.0% (17.1% vs 25.4%; relative risk 0.68; 95% CI, 0.45-1.00) according to definition 2 compared with CMV. No significant between-group differences were observed for death, higher than stage 2 retinopathy of prematurity, stage 2 or higher necrotizing enterocolitis, grade 3 or higher intraventricular hemorrhage, air leak, or hemodynamically significant patent ductus arteriosus. Sensitivity analysis excluding 44 participants who crossed over between treatment groups did not significantly attenuate the estimates.

**Conclusions and Relevance:**

This randomized clinical trial found that elective HFOV reduced the incidence of BPD in preterm infants born at 34 weeks’ gestation or earlier with NARDS compared with CMV. The results of this study suggest that elective HFOV is a promising strategy for preventing BPD in this high-risk population, especially the more severe forms linked to increased long-term morbidity and mortality.

**Trial Registration:**

ClinicalTrials.gov Identifier: NCT03591796.

## Key Points

**Question:**

Does elective high-frequency oscillatory ventilation (HFOV) reduce the incidence of bronchopulmonary dysplasia (BPD) and other neonatal adverse outcomes compared with conventional mechanical ventilation (CMV) among preterm infants diagnosed with neonatal acute respiratory distress syndrome?

**Findings:**

In this randomized clinical trial of 386 preterm infants, elective HFOV reduced the risk of BPD according to the 2001 Eunice Kennedy Shriver National Institute of Child Health and Human Development definition by 24.0% and the risk of BPD according to the 2019 Jensen definition by 32.0% compared with CMV.

**Meaning:**

The results of this study suggest that elective HFOV is a promising strategy for preventing BPD in preterm infants with neonatal acute respiratory distress syndrome, especially the more severe forms associated with increased long-term morbidity and mortality.

## Teaser

This randomized clinical trial compares the effectiveness of elective high-frequency oscillatory ventilation vs conventional mechanical ventilation in reducing the incidence of bronchopulmonary dysplasia among preterm infants with acute respiratory distress syndrome.

## Introduction

The first international consensus definition of neonatal acute respiratory distress syndrome (NARDS) was published in 2017 by De Luca et al.^1^ Among affected neonates, the reported mean gestational age (GA) ranged from 32.3 to 36.4 weeks, with in-hospital mortality rates between 12.6% and 23.8%.^2,3^ These findings underscored that key clinical features of NARDS, including incidence, male predominance, mortality, injury factors, timing of onset, and response to surfactant, are broadly comparable to those observed in pediatric and adult ARDS. However, despite extensive research in adult and pediatric populations, evidence remains insufficient to recommend high-frequency oscillatory ventilation (HFOV) or conventional mechanical ventilation (CMV) as the preferred first-line therapy.^4,5,6,7^

A recent animal study suggested that HFOV may benefit preterm baboons with acute pulmonary dysfunction, typically due to respiratory distress syndrome (RDS), by using low tidal volume, supraphysiologically higher respiratory rate, and lower peak inspiratory pressure to enhance oxygenation and gas exchange.^8^ The team also reported that elective HFOV vs CMV was associated with a modest reduction in bronchopulmonary dysplasia (BPD).^8^ However, randomized clinical trials (RCTs) in humans have yielded inconsistent findings,^7,9,10,11,12^ likely reflecting differences between animal models, in which RDS was induced and treated with surfactant alone, and clinical scenarios, in which preterm birth often involved complex origins requiring both surfactant and antibiotics for placental insufficiency or intrauterine infection. A previous study found that HFOV did not significantly reduce BPD risk compared with CMV in 700 infants born between 24 and 42 weeks with NARDS,^13^ although subgroup analysis suggested benefit in 175 infants born at a GA of 34 weeks or less. Such findings highlight lack of robust evidence for optimizing ventilation strategies in preterm infants born at a GA of 34 weeks or less with NARDS and the need for well-designed trials in this high-risk population.

To address this gap, we designed an RCT to compare elective HFOV with CMV in reducing the incidence of BPD among preterm infants born between 25 weeks 0 days and 34 weeks 6 days and diagnosed with NARDS. We hypothesized that elective HFOV could significantly lower the risk of BPD and improve secondary outcomes, including death, higher than stage 2 retinopathy of prematurity (ROP), stage 2 or higher necrotizing enterocolitis (NEC), grade 3 or higher intraventricular hemorrhage (IVH), and hemodynamically significant patent ductus arteriosus (hsPDA).

## Methods

### Study Design 

This single-center, single-blinded RCT was conducted at a tertiary neonatal intensive care unit at Children’s Hospital of Chongqing Medical University, People’s Republic of China, from August 1, 2019, to December 31, 2023. Eligibility requirements for neonates were as follows: (1) GA between 25 weeks 0 days and 34 weeks 6 days; (2) homogeneous Chinese preterm neonates admitted to the neonatal intensive care unit within 12 hours after birth who were diagnosed with NARDS using the Montreux guidelines^1^ and in stable condition supported by CMV; and (3) stable for 2 hours before randomization (fraction of inspired oxygen [FiO_2_] of ≤0.40, mean airway pressure [MAP] of ≤10-14 cm H_2_O, respiratory rate of ≤40/min, oxygen saturation as measured by pulse oximetry [SpO_2_] of 90%-94%, pH >7.20, PaCO_2 _of ≤60 mm Hg, tidal volume of 5 mL/kg, and >35% hematocrit; these may be evaluated by arterial blood gas analysis). Neonates were not included if any of the following criteria were met: (1) parents’ decision not to participate, (2) major congenital anomalies or chromosomal abnormalities, (3) upper respiratory tract abnormalities, (4) need for surgery before randomization, and (5) higher than grade 2 IVH. The study was terminated if any of the following conditions were met: (1) death, (2) parental decision to withdraw participation, or (3) discharge based on the attending physician’s recommendation. The study was approved by the Ethics Committee of Children’s Hospital of Chongqing Medical University. Written informed consent was obtained before the study. The trial was conducted in accordance with the approved guidelines and regulations of the participating institutions. This study followed the Consolidated Standards of Reporting Trials (CONSORT) reporting guideline. The trial protocol can be found in Supplement 1.

### Allocation and Blinding

After parental consent was obtained, eligible preterm infants initially receiving CMV were randomized to HFOV or CMV using a random-number table and sealed, opaque envelopes (J.L. and L.K.Z.). Due to the nature of the intervention, we were unable to mask invasive supporting strategies from caregivers; however, parents or legal guardians and clinical investigators (Z.F. and Y.S.) were blinded to them. Additionally, statistical analysts (Q.Y., L.C., and L.-J.L.) remained blinded to the group allocations to maintain the integrity of the data analysis.

### Intervention and Control Group Management

#### Intervention Group

Elective HFOV was provided only with piston or membrane oscillators capable of delivering true oscillatory pressure with an active expiratory phase (ie, Acutronic FABIAN-III [Acutronic Medical Systems AG], SLE 5000 [SLE Ltd], LEONI+ [Löwenstein Medical], or Sensormedics 3100A [Vyaire Medical]). Other machines offering high-frequency ventilation were excluded. A lung recruitment maneuver was performed as previously described,^14^ and lung volume was assessed by chest radiography or lung ultrasonography, targeting the right diaphragm at the level of 8th to 9th rib (or 7th-8th rib in case of air leak).^6^ The initial ventilator settings included the following: (1) MAP of 8.0 cm H_2_O (adjustable from 6 to 20 cm H_2_O in 1–cm H_2_O steps); (2) frequency of 10 Hz (adjustable from 8 to 20 Hz in 1-Hz steps); (3) inspiration to expiration ratio fixed at 1:1; (4) amplitude of 20 cm H_2_O (adjustable from 15 to 50 cm H_2_O in 1–cm H_2_O steps); (5) FiO_2_ of 0.30 (adjustable from 0.21 to 1.00 in 0.50 steps); and (6) high-frequency tidal volume (equal to 2.0 mL/kg if used [adjustable from 1.0 to 2.5 mL/kg in 0.1-mL/kg steps]).^7^

#### CMV Group

CMV was delivered by time-cycled, pressure-limited ventilators. Only pressure-regulated volume control is provided by any type of neonatal ventilator. The starting parameters were a respiratory rate of 30/min (adjustable from 10 to 50/min in 2/min steps), inspiratory time of 0.4 second (adjustable from 0.3 to 0.6 second in 0.05-second steps), peak inspiratory pressure of 16 cm H_2_O (adjustable from 6 to 30–cm H_2_O in 1–cm H_2_O steps of 1), positive end expiratory pressure (PEEP) of 6 cm H_2_O (adjustable from 4 to 12 cm H_2_O in 1–cm H_2_O steps), FiO_2_ of 0.30 (adjustable from 0.21 to 1.00 in 5% steps), and tidal volume of 5 mL/kg (adjustable from 3 to 6 mL/K in 0.5-mL/kg steps). Subsequently, ventilator settings were adjusted at the discretion of the attending clinician to maintain an SpO_2 _between 90% and 94%, a PaO_2_ between 50 and 80 mm Hg, a PaCO_2_ between 35 and 60 mm Hg, and a pH between 7.20 and 7.45. The PaO_2_ and PaCO_2_ levels were monitored using arterial blood gas analysis and/or transcutaneous monitoring in both groups.

#### HFOV and CMV Crossover

This study allowed infants who did not respond to their assigned ventilation mode to receive a trial of the alternate mode. Crossover criteria for HFOV-assigned neonates included failure for 3 hours to maintain an SpO_2 _of 90% or higher despite FiO_2 _of 1.0, PaCO_2_ greater than 60 mm Hg for 3 hours, or signs of ventilator-induced cardiac output reduction. Nonresponders to HFOV were switched to CMV. Crossover criteria for CMV-assigned neonates included inability for 3 hours to maintain an SpO_2 _of 90% or greater despite an FiO_2_ of 1.0, a PaCO_2_ greater than 60 mm Hg for 3 hours, or need of a peak inspiratory pressure greater than 30 cm H_2_O to sustain ventilation. Nonresponders to CMV were switched to HFOV.

#### Invasive Ventilation Weaning

The intervention ended at the first successful weaning from invasive ventilation. Extubation requirements were consistent with a previous study (details in the trial protocol [Supplement 1]).^12^ Extubation was followed immediately by the initiation of nasal intermittent positive pressure ventilation. The setting of respiratory parameters of nasal intermittent positive pressure ventilation and subsequent administration was consistent with a previous report.^15^ The patients would be reintubated if they were not improved and needed invasive ventilation.^16^ If the first extubation failed in a preterm infant, the new invasive modes and noninvasive supporting mode of the second extubation were at the discretion of the attending neonatologist.

#### Management and Administration of Medication 

All neonates were continuously monitored for SpO_2_, electrocardiographic signals, heart rate, and respiratory rate. PaCO_2_ was assessed using arterial blood gas analysis and/or transcutaneous monitoring following the guidelines of the Chinese Association of Pediatrics and manufacturer recommendations.^17^ The frequency of blood gas sampling was determined by the attending clinicians. As no specific management guidelines for NARDS were available, surfactant administration followed the European consensus guideline for the management of RDS.^18^

### Primary and Secondary Outcomes

The primary outcome was the incidence of overall BPD, according to the Eunice Kennedy Shriver National Institute of Child Health and Human Development (NICHD) definition in 2001^19^ and the definition by Jensen et al^20^ in 2019, respectively. BPD using the 2001 NICHD definition was diagnosed if oxygen was needed for 28 days after birth. At 36 weeks’ GA, mild BPD will be diagnosed if no oxygen was needed, moderate BPD if less than 30% oxygen was needed, and severe BPD is 30% or more oxygen or noninvasive positive pressure or mechanical ventilation was needed.^19^ BPD using the 2019 Jensen et al^20^ definition was diagnosed according to the mode of respiratory support at 36 weeks’ GA. If infants were discharged from hospital before 36 weeks’ GA, BPD severity was assessed on the basis of the respiratory support administered at discharge. Infants receiving no supplemental respiratory support were divided into no BPD, those treated with nasal cannula (≤2 L/min) as grade 1 BPD, those treated with nasal cannula (>2 L/min) or noninvasive positive airway pressure as grade 2 BPD, and those treated with invasive mechanical ventilation as grade 3 BPD.^20^ The secondary outcomes were major neonatal adverse outcomes, including death, total ventilation duration, air leak,^17^ hsPDA,^21^ greater than stage 2 ROP,^22^ stage 2 or greater NEC,^23^ and greater than grade 2 IVH.^24^

### Statistical Analysis

No previous RCTs were performed comparing HFOV and CMV in preterm infants with NARDS. According to prior RCTs comparing HFOV with CMV in preterm infants, reported differences in outcomes ranged from 4.5% to 37.5% among infants with respiratory insufficiency at GAs of 26 to 30 weeks.^6,25,26,27,28^ To ensure a conservative estimate for our study population, we adopted a median effect size of 13% in BPD incidence between HFOV and CMV treatment. Based on an assumed baseline incidence of 28.7% among preterm neonates born at 34 weeks or less and diagnosed with NARDS in our population,^2^ we estimated that a minimum of 320 neonates (160 per group) would be required to achieve 80% power at a 2-sided *P* < .05. Sample size calculation was performed using PASS software, version 8.0.3 (NCSS LLC).

Normally distributed data are presented as mean (SD) and compared using a 2-tailed *t* test or as median (IQR) and compared using the Mann-Whitney *U* test. Categorical variables were presented as numbers (percentages) and compared using the χ^2^ test or Fisher exact test, as appropriate. Data analyses were conducted according to the intention-to-treat principle, which requires that all randomized participants be included and analyzed based on the treatment group to which they were originally assigned. In our intention-to-treat analysis, modified Poisson regression was used to estimate the relative risk (RR) with 95% CIs for the primary and most secondary outcomes. Cox proportional hazards regression was applied to estimate the hazard ratio (HR) for mortality between groups. Due to crossover cases during the RCT, we conducted sensitivity analyses as follows: (1) reran the regression based on the final treatment received by each group and (2) excluded all crossover participants from the analysis. Additional adjustments were only applied if any prognostic variable was found with significant difference between 2 groups by the end of the trial. All statistical analyses were performed using R, version 4.2.2 (R Foundation for Statistical Computing). A 2-tailed *P* < .05 was considered statistically significant.

## Results

A total of 632 preterm infants were screened. Of these, 222 were excluded, 410 obtained parental initial consent, and 24 withdrew due to parental refusal before randomization, Ultimately, 386 infants (230 male [59.6%] and 156 [40.4%] female; mean [SD] maternal age, 29.9 [4.8] years) were randomized and completed the trial: 181 in the HFOV group and 205 in the CMV group (Figure). During the trial, 24 infants in the HFOV group were switched to CMV, whereas 20 in the CMV group were switched to HFOV. Comparisons of clinical characteristics between crossover cases and their originally assigned groups are provided in eTables 1 and 2 in Supplement 2.

**Figure.**
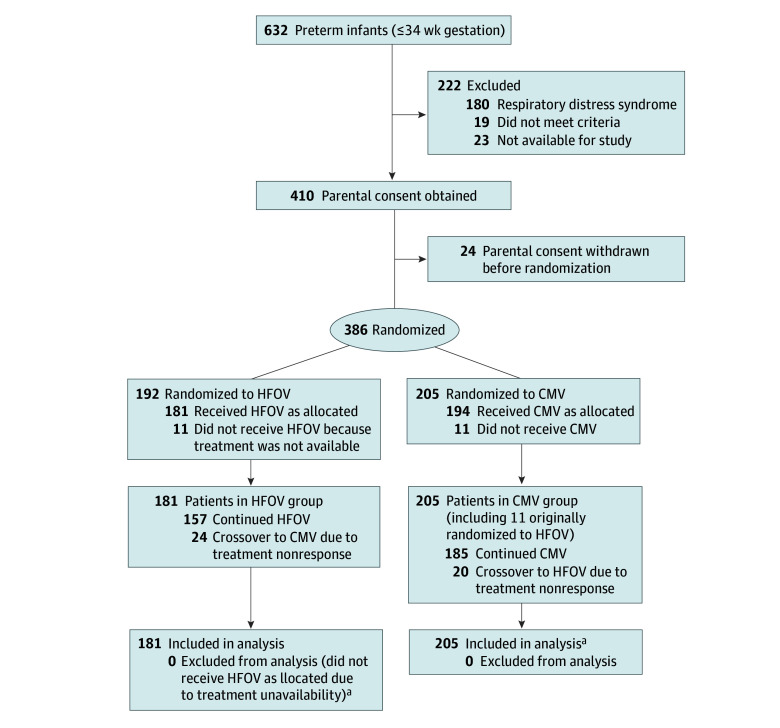
CONSORT Flow Diagram CMV indicates conventional mechanical ventilation; HFOV, high-frequency oscillation ventilation. ^a^Patients who were allocated to HFOV but did not receive it because of unavailability were analyzed as part of the CMV group.

Among all 386 participants, 154 (39.9%) developed BPD using the 2001 NICHD guidelines, including 62 of the 181 (34.3%) in the HFOV group (5 in the HFOV plus volume guarantee subgroup) and 92 of the 205 (44.9%) in the CMV group. The superiority post hoc power calculation based on the actual sample size and observed incidence of BPD between groups indicated that the study achieved a power of 0.70. Most baseline clinical characteristics were similar between groups (Table 1). However, the HFOV group had significantly higher MAP (11.2 vs 10.4 cm H_2_O, *P* < .001) at NARDS diagnosis compared with the CMV group (Table 1). The difference between groups for oxygenation index was not statistically significant (8.30 vs 8.00; *P* = .06).

**Table 1.** Baseline Characteristics of the Study Participants

Characteristic	No. (%) of participants	
	HFOV (n = 181)	CMV (n = 205)
Maternal characteristics at baseline		
Maternal age, mean (SD), y	30.0 (5.0)	29.8 (4.6)
Nulliparous	56 (30.9)	56 (27.3)
Smoking during pregnancy	5 (2.8)	6 (2.9)
Preexisting hypertension or HDP	2 (1.1)	3 (1.5)
Preexisting diabetes or gestational diabetes	2 (1.1)	3 (1.5)
Gestational hypothyroidism	9 (5.0)	18 (8.8)
Use of antenatal corticoids	101 (55.8)	118 (57.6)
Cesarean delivery	48 (26.5)	42 (20.5)
Antepartum hemorrhage	22 (12.2)	20 (9.8)
Premature rupture of the membrane	55 (30.4)	69 (33.7)
Intrahepatic cholestasis of pregnancy	10 (5.5)	7 (3.4)
Chorioamnionitis	6 (3.3)	9 (4.4)
Infant characteristics at baseline		
Sex		
Male	101 (55.8)	129 (62.9)
Female	80 (44.2)	76 (37.1)
Birth weight, mean (SD), g	1610 (546)	1710 (573)
Gestational age at delivery, mean (SD), wk	30.60 (2.42)	31.00 (2.42)
Apgar score at 5 min, median (IQR)	8.00 (7.00-9.00)	8.00 (8.00-9.00)
Age after birth, mean (SD)	4.89 (6.10)	4.81 (6.49)
Time to diagnose NARDS, mean (SD), h	22.60 (6.11)	23.20 (6.87)
Time to diagnose NARDS		
Within 24 h	131 (72.4)	145 (70.7)
Within 24-48 h	50 (27.6)	58 (28.3)
Within 48-72 h	0	2 (1.0)
Baseline blood albumin, mean (SD), g/dL	2.57 (0.53)	2.60 (0.44)
Baseline blood white blood cell count, mean (SD), /µL	11 700 (6960)	11 600 (7160)
Use of antibiotics	179 (98.9)	205 (100)
Use of surfactant	95 (52.5)	96 (46.8)
MAP at NARDS diagnosis, mean (SD), cm H_2_O	11.20 (2.56)	10.40 (1.95)
OI at NARDS diagnosis, median (IQR)	8.30 (6.20-13.60)	8.00 (5.80-10.40)
Severity of NARDS		
Mild NARDS (OI between 4-8)	80 (44.2)	100 (48.8)
Moderate-to-severe NARDS (OI >8)	101 (55.8)	105 (51.2)
Meconium aspiration syndrome	2 (1.1)	6 (2.9)
Pulmonary hemorrhage	43 (23.8)	47 (22.9)
Persistent pulmonary hypertension of newborn	79 (43.6)	92 (44.9)
Early-onset sepsis	112 (61.9)	117 (57.1)

Abbreviations: CMV, conventional mechanical ventilation; HDP, hypertensive disorders of pregnancy; HFOV, high-frequency oscillation ventilation; MAP, mean airway pressure; NARDS, neonatal acute respiratory distress syndrome; OI, oxygenation index.

SI conversion factors: To convert albumin to grams per liter, multiply by 10; white blood cells to ×10^9^/L, multiply by 0.001.

Regarding the primary outcome, infants in the HFOV group demonstrated a 24.0% reduced risk of developing BPD according to the 2001 NICHD definition (34.3% vs 44.9%; RR, 0.76; 95% CI, 0.59-0.98) and a 32.0% reduced risk of developing BPD according to the 2019 Jensen et al^20^ definition (17.1% vs 25.4%; RR, 0.68; 95% CI, 0.45-1.00) compared with the CMV group (Table 2). Sensitivity analyses, including additionally adjusting for postprognostic variable differing between the 2 groups (MAP and oxygenation index at NARDS diagnosis), exclusion of crossover participants, and reclassification of sample size in each group based on final treatment received, confirmed that the beneficial effect of HFOV on reducing overall BPD remained statistically significant (ITT analysis: RR, 0.76; 95% CI, 0.59-0.98 for the 2001 BPD NICHD definition and RR, 0.68; 95% CI, 0.45-1.00 for the 2019 Jensen et al^20^ definition) (Table 3). For secondary outcomes, no significant differences were found between the HFOV and CMV groups (Table 2; eFigure in Supplement 2).

**Table 2.** Modified Poisson Regression and Cox Proportional Hazards Regression for Risk of BPD and Other Outcomes

Study outcome	No. (%) of participants^a^		Estimate (95% CI)^b^
	HFOV (n = 181)	CMV (n = 205)	
Primary outcome			
BPD (2001 NICHD definition)	62 (34.3)	92 (44.9)	0.76 (0.59 to 0.98)
BPD (2019 Jensen et al^20^ definition)	31 (17.1)	52 (25.4)	0.68 (0.45 to 1.00)
Secondary outcomes			
Death	35 (19.3)	34 (16.6)	1.24 (0.77 to 1.99)
BPD (2001 NICHD definition) or death	92 (50.8)	117 (57.1)	0.89 (0.74 to 1.07)
BPD (2019 Jensen et al^20^ definition) or death	63 (34.8)	78 (38.0)	0.92 (0.70 to 1.19)
Ventilation total duration, median (IQR), h	94.1 (49.0-162.0)	73.6 (49.0-163.0)	0.40 (−0.75 to 11.66)
Greater than stage 2 ROP	8 (4.4)	8 (3.9)	1.13 (0.43 to 2.96)
hsPDA	10 (5.5)	6 (2.9)	1.89 (0.70 to 5.00)
Grade 3 or greater IVH	6 (3.3)	11 (5.4)	0.62 (0.23 to 1.64)
Stage 2 or greater NEC	5 (2.8)	6 (2.9)	0.94 (0.29 to 3.04)
Air leak	2 (1.1)	7 (3.4)	0.32 (0.07 to 1.54)

Abbreviations: BPD, bronchopulmonary dysplasia; CMV, conventional mechanical ventilation; HFOV, high-frequency oscillatory ventilation; hsPDA, hemodynamic significant patent ductus arteriosus; IVH, intraventricular hemorrhage; NICHD, Eunice Kennedy Shriver National Institute of Child Health and Human Development; NEC, necrotizing enterocolitis; ROP, retinopathy of prematurity.

^a^ Unless otherwise indicated.

^b^ All estimates are relative risks except for death (hazard ratio) and ventilation total duration (median difference).

**Table 3.** Sensitivity Analysis Results for BPD Risk in the Intervention and Control Groups

Study outcome	No. (%) of participants		RR (95% CI)				
	HFOV (n = 181)	CMV (n = 205)	ITT analysis (n = 386)	Adjusting for MAP at NARDS diagnosis (n = 386)	Adjusting for OI at NARDS diagnosis (n = 386)	Excluding 44 crossover participants (n = 342)	Actual treatment given at study end point (n = 386)
BPD (2001 NICHD definition)	62 (34.3)	92 (44.9)	0.76 (0.59-0.98)	0.77 (0.60-1.00)	0.77 (0.60-0.99)	0.72 (0.54-0.94)	0.73 (0.57-0.95)
BPD (2019 Jensen et al^20^ definition)	31 (17.1)	52 (25.4)	0.68 (0.45-1.00)	0.67 (0.45-1.01)	0.67 (0.45-1.00)	0.56 (0.36-0.89)	0.60 (0.40-0.90)

Abbreviations: BPD, bronchopulmonary dysplasia; CMV, conventional mechanical ventilation; HFOV, high-frequency oscillation ventilation; ITT, intention to treat; MAP, mean airway pressure; NARDS, neonatal acute respiratory distress syndrome; NICHD, Eunice Kennedy Shriver National Institute of Child Health and Human Development; OI, oxygenation index; RR, relative risk.

## Discussion

In this single-center, single-blinded RCT, elective HFOV reduced the risk of BPD according to the 2001 NICHD definition by 24.0% and the risk of BPD according to the 2019 Jensen et al^20^ definition by 32.0% compared with CMV among neonates with NARDS born at a GA of 34 weeks or less. However, HFOV did not significantly affect mortality or other adverse neonatal outcomes, including BPD or death, total ventilation duration, greater than stage 2 ROP, stage 2 or greater NEC, grade 3 or greater IVH, or hsPDA.

Our study was consistent with the meta-analyses by Cools et al^10^ and Yu et al^27^ as well as several RCTs.^14,25,26^ On the contrary, other studies^6,27,29,30,31,32,33^ found no significant differences, aligning with another meta-analysis.^9^

The inconsistent findings between HFOV and CMV may largely reflect the heterogeneity of respiratory distress diagnoses. A key challenge lies in the substantial clinical overlap between RDS and NARDS, in terms of origin, pathophysiology, diagnosis, and treatment.^34^ This overlap complicates the reliable exclusion of RDS in neonates suspected of having NARDS. In a previous study^2^ on NARDS, cumulative incidences of NARDS reached 65.6%, 86.7%, and 94.1% within 1, 2, and 3 days after birth, respectively. There is up to a 30.3% co-occurrence between RDS and NARDS. These findings highlight the difficulty of distinguishing RDS from NARDS within the first 24 hours of life, a challenge compounded by the lack of a precise definition of RDS.^35^ Clarifying this distinction is critical for improving diagnostic accuracy and enhancing the applicability and clinical utility of the RDS guideline.

According to the European Management Guideline of RDS in 2023, no exact definition of RDS is provided, which complicates differential diagnosis between RDS and NARDS. The guidelines also note that management now emphasizes preemptive surfactant treatment guided by clinical assessment of breathing effort and oxygen requirement.^34^ In this study, preterm infants with RDS were excluded based on the NARDS definition by De Luca et al,^1^ which requires respiratory distress within the first 24 hours of life and a complete, sustained, and prompt response to surfactant or lung recruitment. Thus, only infants with NARDS were included, ensuring high cohort homogeneity.

Another major source of heterogeneity lies in the criteria used for surfactant administration and readministration, which varied considerably across studies. Some trials^6,26,27,29,30,31^ administered surfactant to all enrolled infants. Others used specific thresholds: Sun et al^14^ used a criterion of PaO_2_/FiO_2 _ratio of less than 200 after 2 hours of ventilation; Ogawa et al^32^ relied on clinical diagnosis of RDS; Rettwitz-Volk et al^36^ required chest radiographic evidence of grade II RDS and an FiO_2 _less than 0.60; and Plavka et al^33^ applied detailed FiO_2 _and pulmonary artery wedge pressure thresholds based on birth weight (eTable 3 in Supplement 2). The use of surfactant in our trial was administered according to the European Consensus Guidelines for RDS, which has not been specified in NARDS guidelines. Due to the RCT design, we believed that both groups were balanced in the benefit of surfactant use. However, the optimal dosing strategy and reuse parameters for surfactant therapy in NARDS warrant for further investigation.

HFOV reduces airway resistance and the work of breathing, lowers lung inflammatory markers, and improves oxygenation, lung compliance, and alveolar development.^4,6,7,8,9,10^ However, the exact mechanism by which HFOV improves efficacy is not fully known. Thus, a better understanding of its exact actions is needed in future studies. One of the possible mechanisms by which HFOV works is that RDS is a primarily restrictive disease of the lung, with less or no lung inflammation, and the use of surfactant and/or noninvasive or invasive ventilation is the cause-specific treatment. In contrast, NARDS is restrictive and obstructive in the acute phase, with high inflammation markers in the lungs. HFOV and CMV are not cause-specific treatments; therefore, HFOV has advantages over CMV, including improved lung ventilation and shorter duration of mechanical ventilation and oxygen use.^37^

### Limitations

Several limitations merit consideration. First, this was a single-center pilot trial conducted in Chinese neonates born at 34 weeks or earlier, which may limit generalizability; multicenter trials and external validation in more diverse populations are needed. Second, the broad GA at delivery ranged between 25 and 34 weeks, which could add to heterogeneity because the incidence of BPD in more immature infants is strongly influenced by comorbidities and their interactions. In addition, it makes no sense to look for BPD in neonates born after 28 weeks. Third, as aforementioned, there is no established guideline for surfactant use in NARDS; in this study, administration even followed RDS protocols, which may not fully reflect NARDS-specific pathophysiology. Fourth, optimizing lung volume based on rib count on chest radiography is likely imprecise, and ultrasonography was not used to assess the lung volume; however, such an effect was balanced by the RCT design in 2 groups. Future studies should incorporate ultrasonography for more accurate assessment. Fifth, clinicians were not blinded when adjusting CMV and HFOV settings, which could introduce considerable variability. Sixth, the post hoc power calculation (0.70) suggests that the study may have been underpowered to detect differences in secondary outcomes. Further research, including stratified analyses, refined diagnostic criteria, and multicenter trials, is warranted to confirm and extend these findings.

## Conclusions

This study found that, among preterm infants receiving ventilatory support who were born at a GA of 34 weeks or less, elective HFOV reduced the incidence of BPD. These findings suggest that elective HFOV may be a promising strategy for preventing BPD in this high-risk population, especially the more severe forms linked to increased long-term morbidity and mortality.

## Notes

Supplement 1.
Trial Protocol

Supplement 2. **eFigure. **Hazard ratio of HFOV group in death compared with CMV group **eTable 1. **The baseline and study endpoint characteristics of mothers and neonates between the selective high frequency oscillation ventilation and conventional mechanical ventilation groups only for cross-over cases, according to the original randomized assignment **eTable 2. **The baseline and study endpoint characteristics of mothers and neonates between the selective high frequency oscillation ventilation and conventional mechanical ventilation groups only for cross-over cases, according to the original randomized assignment **eTable 3.** Details on surfactant use among studies

Supplement 3.
Data Sharing Statement
