# Errors in Abstract, Results, Table 1, Table 2, Table 3, Discussion, and Visual Abstract

**DOI:** 10.1001/jamanetworkopen.2026.22801

**Published:** 2026-06-15

In the Original Investigation titled “High-Frequency Oscillation vs Mechanical Ventilation for Neonatal Acute Respiratory Distress Syndrome: A Randomized Clinical Trial,”^1^ published March 9, 2026, there were errors in the Abstract, Results, Table 1, Table 2, Table 3, Discussion, and Visual Abstract. In Table 1, units of measure for “Apgar score at 5 min” and “OI at NARDS diagnosis” should have been labeled as being reported in median (IQR); “Baseline blood albumin,” “Baseline blood white blood cell count,” and “MAP at NARDS diagnosis” should have been labeled as reported in mean (SD). In Table 2, the bronchopulmonary dysplasia (2001 NICHD definition) primary outcome estimate (95% CI) should have been 0.76 (0.59 to 0.98); for secondary outcomes, estimate (95% CI) values should have been 0.62 (0.23 to 1.64) for grade 3 or greater IVH, 0.94 (0.29 to 3.04) for stage 2 or greater NEC, and 0.32 (0.07 to 1.54) for air leak; ventilation total duration should have been listed as reported in median (IQR); footnote b should have ended with “ventilation total duration (median difference).” In Table 3, bronchopulmonary dysplasia (2001 NICHD definition) study outcome relative risk (95% CI) values should have been listed as 0.76 (0.59-0.98) for ITT analysis, 0.77 (0.60-1.00) for adjusting for MAP at NARDS diagnosis, 0.77 (0.60-0.99) for adjusting for OI at NARDS diagnosis, 0.72 (0.54-0.94) for excluding 44 crossover participants, and 0.73 (0.57-0.95) for actual treatment given at study end point. All corrections made in Tables 2 and 3 were also made in applicable text in the Abstract, Results, and Visual Abstract. Additionally, in the Abstract, Results, and Discussion, the primary outcome should have been reported as infants in the HFOV group having a 24.0% reduced risk of developing bronchopulmonary dysplasia according to the 2001 NICHD definition. This article has been corrected.^1^
