# Supplementary material for: High-Frequency Oscillation vs Mechanical Ventilation for Neonatal Acute Respiratory Distress Syndrome: A Randomized Clinical Trial
Source: JAMA Netw Open. 2026 Mar 9;9(3):e260268. doi: 10.1001/jamanetworkopen.2026.0268 (PMC12973098; doi:10.1001/jamanetworkopen.2026.0268)
Supplement: Supplement 1. — Trial Protocol [file jamanetwopen-e260268-s001.pdf]

Supplemental Online Content

Li J, Liu K, Yang Q, et al. High-frequency oscillation vs mechanical ventilation for neonatal acute respiratory distress syndrome: a randomized clinical trial. *JAMA Netw Open.* 2026;9(3):e260268. doi:10.1001/jamanetworkopen.2026.0268

eFigure. Hazard ratio of HFOV group in death compared with CMV group

eTable 1. The baseline and study endpoint characteristics of mothers and neonates between the selective high frequency oscillation ventilation and conventional mechanical ventilation groups only for cross-over cases, according to the original randomized assignment

eTable 2. The baseline and study endpoint characteristics of mothers and neonates between the selective high frequency oscillation ventilation and conventional mechanical ventilation groups only for cross-over cases, according to the original randomized assignment

eTable 3. Details on surfactant use among studies

This supplemental material has been provided by the authors to give readers additional information about their work.

© 2026 Li J et al. *JAMA Network Open.*

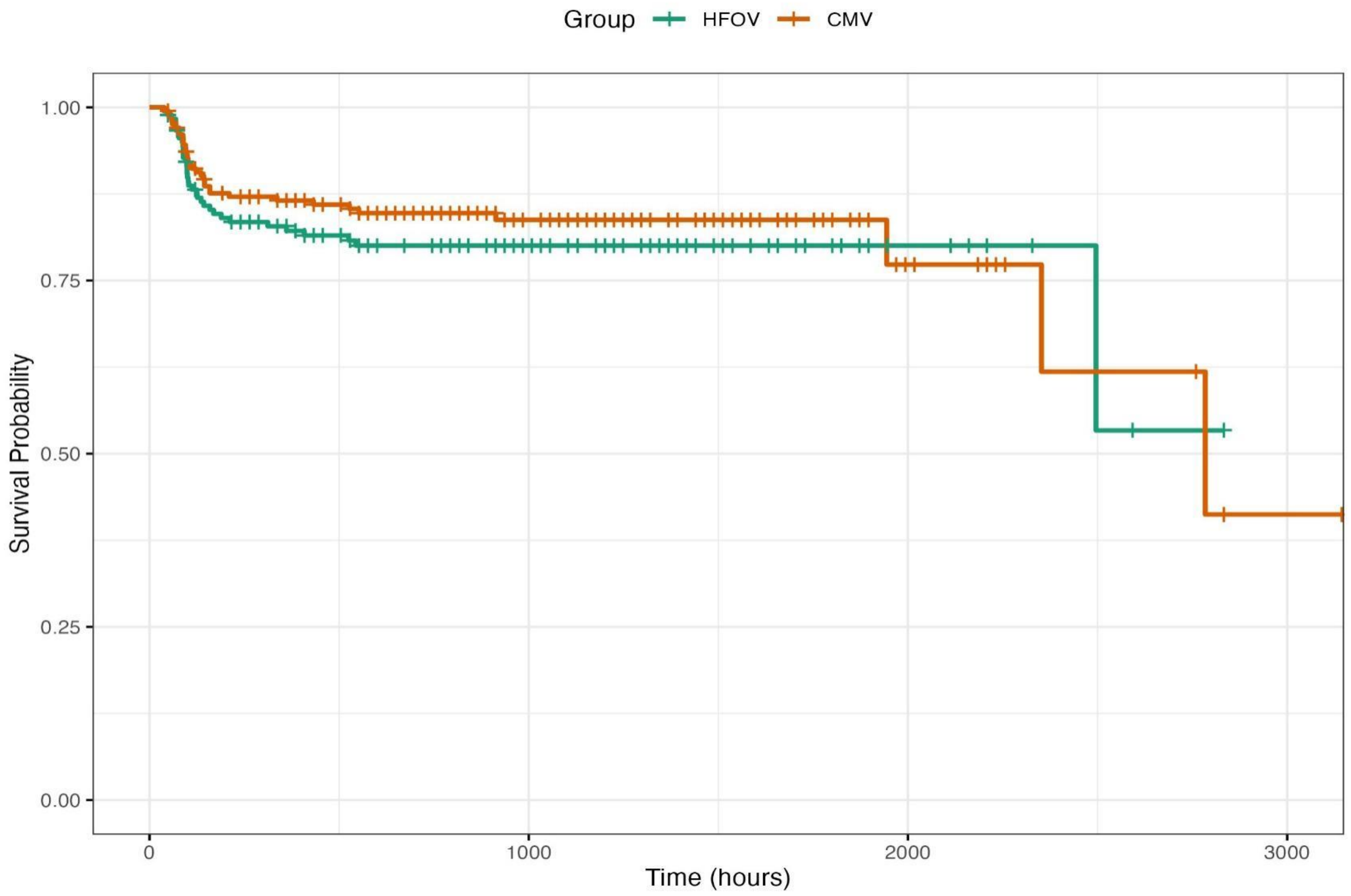

eFigure. Hazard ratio of HFOV group in death compared with CMV group.

© 2026 Li J et al. *JAMA Network Open.*

eTable 1. The baseline and study endpoint characteristics of mothers and neonates between the selective high frequency oscillation ventilation and conventional mechanical ventilation groups only for cross-over cases, according to the original randomized assignment

**Characteristics and Outcomes**	**Always in HFOV (n = 157) Mean (SD), Median (IQR), or n (%)**	**Move to CMV later (n = 24) Mean (SD), Median (IQR), or n (%)**	**p**
Maternal characteristics at baseline			
Maternal age, years	29.8 (4.83)	31.1 (5.99)	0.32
Nulliparous, yes	51 (32.5%)	5 (20.8%)	0.36
Smoking during pregnancy, yes	4 (2.5%)	1 (4.2 %)	0.52
Pre-existing hypertension or HDP, yes	2 (1.3%)	0 (0%)	0.99
Pre-existing diabetes or GDM, yes	1 (0.6%)	1 (4.2%)	0.25
Gestational hypothyroidism, yes	9 (5.7%)	0 (0%)	0.61
Use of antenatal corticoids, yes	89 (56.7%)	12 (50.0%)	0.66
Caesarean, yes	44 (28.0%)	4 (16.7%)	0.32
Antepartum hemorrhage, yes	16 (10.2%)	6 (25.0%)	0.08
PROM (n, %)	48 (30.6%)	7 (29.2%)	0.99
Gestational age at delivery, weeks	30.6 (2.41)	31.1 (2.54)	0.36
ICP, yes	9 (5.7%)	1 (4.2%)	0.99
Chorioamnionitis	4 (2.5%)	2 (8.3%)	0.18
Infant characteristics at baseline			
Infant sex, boys	92 (58.6%)	9 (37.5%)	0.09
Birth weight, gram	1590 (543)	1700 (567)	0.38
Apgar score at 5 min	8.00 (2.00)	8.00 (2.25)	0.37
Age after birth, hour	4.99 (6.04)	4.21 (6.58)	0.12
Time to diagnose NARDS, hours	22.6 (6.10)	22.4 (6.37)	0.90
Time to diagnose NARDS, category			0.64

© 2026 Li J et al. *JAMA Network Open.*

Within 24 hours， yes	112 (71.3%)	19 (79.2%)	
within 24-48 hours， yes	45 (28.7%)	5 (20.8%)	
Within 48-72 hours， yes	0 (0%)	0 (0%)	
Baseline blood albumin, g/L	25.8 (5.10)	25.3 (6.54)	0.69
Baseline blood white blood cell count, $*10^9$/L	12.0 (7.18)	9.71 (4.99)	0.06
Use of antibiotics, yes	156 (99.4%)	23 (95.8%)	0.62
Use of Surfactant, yes	83 (52.9%)	12 (50.0%)	0.97
Mean airway pressure (MAP) at diagnosis of NARDS, cmH_2O	11.0 (2.41)	12.3 (3.25)	0.09
Oxygenation Index (OI) at diagnosis of NARDS	8.00 (6.90)	10.2 (11.1)	**0.02**
Severity of NARDS			
Mild NARDS (OI between 4-8)	75 (47.8%)	5 (20.8%)	**0.02**
Moderate-to-severe NARDS (OI >8)	82 (52.2%)	19 (79.2%)	
MAS, yes	1 (0.6%)	1 (4.2%)	0.25
PH, yes	34 (21.7%)	9 (37.5%)	0.12
PPHN, yes	66 (42.0%)	13 (54.2%)	0.28
EOS, yes	99 (63.1%)	13 (54.2%)	0.50

Abbreviations: HFOV: high frequency oscillation ventilation; CMV: conventional mechanical ventilation; NARDS: neonatal acute respiratory distress syndrome; MD: mean difference; RR: relative risk; PROM: premature rupture of the membrane; GDM: gestational diabetes mellitus; ICP: intrahepatic cholestasis of pregnancy; PPHN: persistent pulmonary hypertension of newborn; MAS: meconium aspiration syndrome; PH: pulmonary hemorrhage; EOS: early onset sepsis; BPD, bronchopulmonary dysplasia; NEC, necrotizing enterocolitis; PDA, patent ductus arteriosus; ROP, retinopathy of prematurity; IVH, intraventricular hemorrhage; PVL, periventricular leukomalacia. LOS, late onset sepsis.

© 2026 Li J et al. *JAMA Network Open.*

eTable 2. The baseline and study endpoint characteristics of mothers and neonates between the selective high frequency oscillation ventilation and conventional mechanical ventilation groups only for cross-over cases, according to the original randomized assignment

**Characteristics and Outcomes**	**Always in CMV (n = 185) Mean (SD), Median (IQR), or n (%)**	**Move to HFOV later (n = 20) Mean (SD), Median (IQR), or n (%)**	**p**
Maternal characteristics at baseline			
Maternal age, years	29.9 (4.70)	29.2 (3.57)	0.39
Nulliparous, yes	49 (26.5%)	7 (35.0%)	0.58
Smoking during pregnancy, yes	5 (2.7%)	1 (5.0%)	0.46
Pre-existing hypertension or HDP, yes	3 (1.6%)	0 (0%)	0.99
Pre-existing diabetes or GDM, yes	3 (1.6%)	0 (0%)	0.99
Gestational hypothyroidism, yes	15 (8.1%)	3 (15.0%)	0.39
Use of antenatal corticoids, yes	111 (60.0%)	7 (35.0%)	0.05
Caesarean, yes	38 (20.5%)	4 (20.0%)	0.99
Antepartum hemorrhage, yes	18 (9.7%)	2 (10.0%)	0.99
PROM (n, %)	61 (33.0%)	8 (40.0%)	0.70
Gestational age at delivery, weeks	31.1 (2.32)	29.6 (2.95)	0.03
ICP, yes	6 (3.2%)	1 (5.0%)	0.99
Chorioamnionitis	6 (3.2%)	3 (15.0%)	**0.05**
Infant characteristics at baseline			
Infant sex, boys	119 (64.3%)	10 (50.0%)	0.31
Birth weight, gram	1730 (559)	1460 (653)	0.08
Apgar score at 5 min	8.00 (1.00)	8.50 (1.00)	0.88
Age after birth, hour	4.82 (6.43)	4.75 (7.16)	0.49
Time to diagnose NARDS, hours	23.3 (7.03)	22.5 (5.26)	0.55
Time to diagnose NARDS, category			0.99

© 2026 Li J et al. *JAMA Network Open.*

Within 24 hours, yes	131 (70.8%)	14 (70.0%)	
within 24-48 hours, yes	52 (28.1%)	6 (30.0%)	
Within 48-72 hours, yes	2 (1.1%)	0 (0%)	
Baseline blood albumin, g/L	26.1 (4.32)	24.9 (5.02)	0.32
Baseline blood white blood cell count, $*10^9/L$	11.9 (7.35)	8.66 (4.19)	**0.006**
Use of antibiotics, yes	185(100%)	20 (100%)	0.99
Use of Surfactant, yes	83 (44.9%)	13 (65.0%)	0.14
Mean airway pressure (MAP) at diagnosis of NARDS, cmH_2O	10.3 (1.91)	10.8 (2.31)	0.43
Oxygenation Index (OI) at diagnosis of NARDS	7.60 (4.20)	9.70 (5.78)	**0.019**
Severity of NARDS			0.10
Mild NARDS (OI between 4-8)	94 (50.8%)	6 (30.0%)	
Moderate-to-severe NARDS (OI >8)	91 (49.2%)	14 (70.0%)	
MAS, yes	6 (3.2%)	0 (0%)	0.99
PH, yes	39 (21.1%)	8 (40.0%)	0.09
PPHN, yes	76 (41.1%)	16 (80.0%)	**0.002**
EOS, yes	103 (55.7%)	14 (70.0%)	0.32

Abbreviations: HFOV: high frequency oscillation ventilation; CMV: conventional mechanical ventilation; NARDS: neonatal acute respiratory distress syndrome; MD: mean difference; PROM: premature rupture of the membrane; GDM: gestational diabetes mellitus; ICP: intrahepatic cholestasis of pregnancy; PPHN: persistent pulmonary hypertension of newborn; MAS: meconium aspiration syndrome; PH: pulmonary hemorrhage; EOS: early onset sepsis.

© 2026 Li J et al. *JAMA Network Open.*

eTable 3. Details on surfactant use among studies

Author	Type of surfactant	First dose of surfactant	Administration of surfactant	Criteria of surfactant	Criteria of re-administration of surfactant
Sun et al 2014	Curosurf	200 mg/kg	in-line catheters	PaO2/FiO_2 was < 200 after 2 hours of ventilation	PaO2/FiO_2 was < 200
Salvo et al 2012	Curosurf	200 mg/kg	endotracheal tube	all infants received	FiO_2 ≥40% (100 mg/kg)
Courtney et al 2012	Not mentioned	Not mentioned	In-line catheters	Before study entry	2nd/3rd doses if infants remained intubated with $FiO_2 \geq 0.30$<br>The 4th dose at the discretion of the attending doctor.
Dani et al 2006	Curosurf	200 mg/kg	tracheal tube	All infants received	An dose of surfactant (100 mg/kg) 12 hr later if an infant still required FiO_2 >0.30
Schreiber et al 2003	Survanta	Not mentioned	Not mentioned	all infants received	Not mentioned
Moriette et al 2001	Curosurf	200 mg/kg	Not mentioned	all infants received	PO_2/FiO_2 < 200 (100 mg/kg)
Durand et al 2001	Survanta	Not mentioned	Ballard Multi-Access Catheter	All infants received	Not mentioned
Plavka et al 1999	Alveofact	50 mg/kg	Not mentioned	HFOV: FiO_2>0.35 or PAwDP ≥12 in infant weigh ≥ 1 kg; PAwDP >10 in infant weigh < 1 kg or CMV: FiO_2≥0.35	HFOV: PA wDP increased by ≥15% and in both groups if the FiO_2 ≥0.35 or increased > 15%
Rettwitz-Volk et al 1998	Survanta	100 mg/kg	Not mentioned	chest x-ray showed RDS grade II and	Not mentioned

© 2026 Li J et al. *JAMA Network Open.*

				$FiO_2 > 0.60$	
Gerstmann et al 1996	Survanta and alveofact	100 mg/kg	Not mentioned	All infants received at least one dose	$Pa/AO_2 < 0.5$
Ogawa et al 1993	bovine surfactant	Not mentioned	Not mentioned	clinical diagnosis of RDS	Not mentioned

© 2026 Li J et al. *JAMA Network Open.*
