# Supplementary material for: High-Frequency Oscillation vs Mechanical Ventilation for Neonatal Acute Respiratory Distress Syndrome: A Randomized Clinical Trial
Source: JAMA Netw Open. 2026 Mar 9;9(3):e260268. doi: 10.1001/jamanetworkopen.2026.0268 (PMC12973098; doi:10.1001/jamanetworkopen.2026.0268)
Supplement: Supplement 2. — eFigure. Hazard ratio of HFOV group in death compared with CMV group eTable 1. The baseline and study endpoint characteristics of mothers and neonates between the selective high frequency oscillation ventilation and conventional mechanical ventilation groups only for cross-over cases, according to the original randomized assignment eTable 2. The baseline and study endpoint characteristics of mothers and neonates between the selective high frequency oscillation ventilation and conventional mechanical ventilation groups only for cross-over cases, according to the original randomized assignment eTable 3. Details on surfactant use among studies [file jamanetwopen-e260268-s002.pdf]

Chongqing Health Centre for Women and Children Chongqing Medical University

Study Title: Invasive Ventilation for Neonates With Acute Respiratory Distress Syndrome (ARDS)

PI: Long Chen, MD PhD, Senior Consultant, Chongqing Health Centre for Women and Children, Chongqing Medical University

Protocol version: 1.0

Protocol date: 1 June 2019

Background

The use of elective high frequency oscillatory ventilation (HFOV) compared with conventional mechanical ventilation (CMV) has theoretical advantage in preterm infants with respiratory distress syndrome (RDS). Animal study also proves that, comparing with CMV, HFOV improves early lung function with sustained improvement in pulmonary mechanics out to 28 d, and has less pulmonary inflammation in the recovery phase of RDS. However, several clinical randomized controlled trials (RCTs) and meta-analyses comparing elective HFOV with CMV have shown inconsistent results. Meta-analyses indicate that heterogeneity is existed in study design, including baseline characteristics and outcomes measures.

Further study found that these RCTs above were all performed in the pre-neonatal acute respiratory distress syndrome (nARDS) era. We therefore speculate that a key heterogeneity may be the included criteria, and perinatal nARDS might be considered as RDS in the pre-nARDS era. Actually, these preterm infants in the two meta-analyses were diagnosed with acute pulmonary dysfunction, of which some were due to RDS, and the others were not detailed. A mixture of different ratio of RDS and nARDS in these studies may result in the inconsistent results. To date, there was no RCT comparing selective HFOV with CMV in preterm infants with perinatal nARDS.

The purpose of the present study was to explore whether selective HFOV was associated with less BPD as compared with CMV in preterm infants with perinatal nARDS.

Patients and Methods

Standard protocol approval, registration, and patient consent.

The study was approved by the Ethics Committee of Children's Hospital of Chongqing Medical University (project approval number:2018117) and registered at http://www.clinicaltrials.gov. (ID: NCT03591796) (the registration date: Nov-20, 2018). The registration name: Invasive Ventilation for Neonates With Acute Respiratory Distress Syndrome(ARDS). It was from a prospective protocol, and informed parental written consents were obtained prior to study. The trial was

performed in accordance with the approved guidelines and regulations of the participating institutions.

Study Design and Participants.

The RCT was conducted in two tertiary neonatal intensive care unit (NICU) from Aug, 2019 to June 2022, Children's Hospital of Chongqing Medical University, China and Chongqing Health Center for Women and Children, China. Eligibility requirements for neonates: (1) gestational age (GA) was less than or equal to 34 weeks; (2) preterm neonates were admitted to NICU within 24 hours after birth, diagnosed with perinatal nARDS and stable supported by CMV; (3) Stabilization before randomization: $FiO_2 \leq 0.40$, Paw ≤10-14 cmH_2O, ≤ 40 bpm of respiratory rate, 90%-94% of SpO_2, pH > 7.20, $PaCO_2 \leq 60$ mmHg and > 35% of hematocrit (these may be evaluated by arterial blood gas analysis).

Neonates with at least one of the following criteria are not eligible for the study: (1) parents' decision not to participate; (2) major congenital anomalies or chromosomal abnormalities; (3) upper respiratory tract abnormalities; (4) need for surgery; (5) more than Grade 2 of intraventricular hemorrhage (IVH).

Allocation and Blinding

After documenting parental consent, the included preterm infants with CMV were randomly assigned to either HFOV or CMV using a table of random numbers and sealed opaque envelopes when they were eligible. C.L. was in possession of envelopes and developed the envelopes, L.KZ. guided allocation and opened envelopes. Blinding towards caregivers was not possible due to the nature of the intervention. Blinding to the preterm infants made no sense. However, the outcomes' assessor (Q.HB and S.Y.) would be blinded, because outcomes of endpoint would be recorded by T.Q., who was not involved in the preterm infants' care. L.F. performing the final statistical analyses was also blinded to the group allocation.

Study Intervention

Selective HFOV +/- volume guarantee (VG): Selective HFOV will only be provided with piston/membrane oscillators able to provide a real oscillatory pressure with active

expiratory phase (that is, Acutronic FABIAN-III, SLE 5000, Loweinstein Med LEONI+, Sensormedics 3100A). Other machines providing high frequency ventilations will not be included. The lung recruitment maneuver was performed as previously described, and lung volume was controlled by chest radiography/lung ultrasound to keep the right diaphragm at the level of 8-9 rib, 7-8 rib if airleak. The starting parameters were mean airway pressure of 8.0 cm of water (subsequent regulation range, 6-20, in step of 1 cm H_2O), frequency of 10 Hz (subsequent regulation range, 8-20, in step of 1 Hz), The ratio of inspiration to expiration fixed at 1:1, the amplitude of 20 (subsequent regulation range, 15-50, in steps of 5 cm H_2O), FiO_2 of 30% (subsequent regulation range, 21-100, in step of 5 cm H_2O) and high-frequency tidal volume (VThf) equaled 2.0 ml/kg if used (subsequent regulation range, 1.0-2.5, in step of 0.1 ml/kg/time). Subsequently, ventilator settings were adjusted at the discretion of the attending clinician to maintain a SpO_2 between 90%-95%, a PaO_2 between 50 and 80 mm Hg, a $PaCO_2$ between 35 and 60 mm Hg (PO_2 and PCO_2 levels were adjusted by transcutaneous monitors) and a PH between 7.20 and 7.45.

CMV: CMV was delivered by time-cycled, pressure-limited ventilators. Only pressure regulated volume control (PRVC) will be provided by any type of neonatal ventilator. The starting parameters were respiratory rate (RR) of 30 per minute (subsequent regulation range, 10-50, in steps of 2), inspiratory time(Ti) of 0.4 second (subsequent regulation range, 0.3-0.6, in steps of 0.05), peak inspiratory pressure (PIP) of 16 cmH_2O (subsequent regulation range, 6-30, in steps of 1), positive end-expiratory pressure (PEEP) of 6 cmH_2O (subsequent regulation range, 4-12, in step of 1) and FiO_2 of 30% (subsequent regulation range, 21-100, in step of 5 cm H_2O), tidal volume of 5ml/kg (subsequent regulation range, 3-6, in step of 0.5 ml/kg/time). Subsequently, ventilator settings were adjusted at the discretion of the attending clinician to maintain a SpO_2 between 90%-95%, a PaO_2 between 50 and 80 mm Hg and a $PaCO_2$ between 35 and 60 mm Hg (PO_2 and PCO_2 levels were adjusted by transcutaneous monitors) and a PH between 7.20 and 7.45.

The crossover to HFOV or CMV

The present study also covered a crossover design which supplied the included infants failing to respond to the assigned mode of ventilation with a trial of the alternate mode of ventilation. Analysis of results was done according to the neonate's original group assignment.

Crossover criteria for neonates assigned to HFOV were failure for 3 hours to maintain an SpO2 $\geq$ 50 mmHg despite receiving a FiO_2 of 1.0, or failure for 3 hours to maintain a $PaCO_2$ $\leq$ 60 mmHg, or evidences of a ventilator-induced cardiac output decrease. Neonates who did not respond to CMV were returned to HFOV.

Crossover criteria for neonates assigned to CMV were failure for 3 hours to maintain an SpO_2 $\geq$ 50 mmHg despite receiving a FiO_2 of 1.0, or failure for 3 hours to maintain an PaCO2 $\leq$ 60 mmHg, or requiring > 30 cm H_2O of PIP to maintain adequate ventilation. Neonates who did not respond to HFOV were returned to CMV.

Weaning from invasive ventilation

Extubation requires fulfilling of all the following criteria: (1). Having received at least one loading dose of 20 mg/kg and 5-10 mg/kg daily maintenance dose of caffeine citrate; (2). pH > 7.20 $PaCO_2$ $\leq$55 mmHg (these may be evaluated by arterial blood gas analysis; (3). Paw $\leq$ 6 cmH_2O; (4). FiO_2 $\leq$ 0.30; (5). sufficient spontaneous breathing effort, as per clinical evaluation;(6).a hematocrit exceeding 35%.[21] When a neonate had fulfilled the extubation criteria, gentle intratracheal and upper airways suction would then be done and nasal intermittent positive pressure ventilation would be started immediately.

Administration of Caffeine citrate, exogenous surfactant replacement, inhaled Nitric Oxide (iNO) and postnatal glucocorticoids.

Caffeine citrate: These preterm infants received caffeine citrate at a loading dose of 20 mg/kg and a maintenance dose of 5-10 mg/kg/d until 34 weeks of gestational age.

Exogeous surfactant replacement: No management guideline of nARDS was available. Therefore, the administration of surfactant was based on the European consensus guideline for the management of RDS. When the neonates were admitted to the NICU and had fulfilled the entry criteria, surfactant (Curosurf, Chiesi Pharmaceuticals, Parma, Italy; or Calsurf, Double-Crane Pharmaceutical Co., Ltd,

Beijing, China) was administered according to the manufacture's instruction as a rescue treatment if an infant needed $FiO_2 > 0.30$ or invasive ventilation to maintain the targeted SpO_2 with 90%-94%. The intervals of surfactant administration were 6 to 12 hours.

iNO: iNO was enforced if SpO_2 was less than 80% after $FiO_2 > 60\%$ or diagnosed with persistent pulmonary hypertension of newborn (PPHN). These preterm infants received iNO at a loading dose of 20ppm for 24 h and a maintenance dose of 5-10 ppm for the next six days.

postnatal glucocorticoids

Infants were ventilator dependent after the first week after birth were eligible. After written informed consent was obtained, infants were administrated twice-daily doses of a 10-day tapering course of dexamethasone of 0.15 mg/kg per day for 3 days, 0.10 mg/kg per day for 3 days, 0.05 mg/kg per day for 2days, and 0.02 mg/kg per day for 2 days. A total dose of 0.89 mg/kg over 10 days.

Eligibility criteria for re-intubation.

The subjects would be re-intubated if they were not improved and needed invasive ventilation. The criteria for re-intubation and mechanical ventilation were one of the following conditions reached: 1. Cardio-respiratory arrest or any types of pulmonary hemorrhage; 2. Two hours of respiratory acidosis with $PaCO_2 > 70$ mmHg and pH < 7.2; 3. Two hours of hypoxia with $PO_2 < 50$ mmHg, under 0.6 of FiO_2 and maximal pressures given of 16 cm H_2O; 4. Apnea 3 times per hour and heart rate less than 100/min; 5. Requiring mask ventilation in any case; 6. Persistent low blood pressure without responsive to liquid resuscitation and vasoactive agents. If a preterm infants failed to the first extubation, the re-needed invasive modes and noninvasive supporting mode of the second extubation was at the discretion of the attending neonatologist.

Termination of the study

The study would end if one of the following conditions was reached: 1. Death; 2. Parents' decision not to continue the participation; 3. Discharge according to doctors' suggestions.

The primary and secondary outcomes.

The primary outcomes will be: (1) the incidence of BPD, according to the NICHD definition in 2001. and definition of BPD in 2019. BPD severity in 2019 was diagnosed according to the mode of respiratory support at a 36 weeks' GA. If a infant was discharged from hospital before 36 weeks' GA, BPD severity was assessed according to respiratory support used at discharge. Infants without supplemental respiratory support were divided into no BPD, those treated with nasal cannula (≤ 2 L/min) as grade 1 BPD, those treated with nasal cannula (> 2 L/min) or noninvasive positive airway pressure as grade 2 BPD and those treated with invasive mechanical ventilation as grade 3 BPD. In the present study, the BPD severity in 2019 was an additional assessment measure and no data of flow rate were available. Therefore, Grades 1 and 2 BPD were combined into a single severity level.

The secondary outcomes were to assess the outcomes between the two groups, including the incidences of BPD or death, death, air leak (pneumothorax and/or pneumomediastinum), haemodynamically significant patent ductusarteriosus (hsPDA), retinopathy of prematurity (ROP) > 2nd stages, necrotizing enterocolitis (NEC) ≥ 2nd stages, and IVH > 2nd grades.

Monitoring and treatments

All neonates will be continuously monitored for SpO_2, ECG, heart and respiratory rate. $PaCO_2$ will be monitored using arterial blood gas analysis and/or transcutaneous monitors according to the Chinese Association of Pediatrics guidelines and the manufacturer's recommendations. Frequency of blood gas analysis will be decided by the attending clinicians. Furthermore, the following treatments or tests will be provided: 1). Placement of umbilical central venous catheter and/or peripherally inserted central venous lines. 2). Ultrasound was used to assess cardiac morphology, pulmonary pressures and PDA within the first 24 hours of life and subsequently repeated every week until normal/leaving hospital. 3) Cerebral ultrasound was assessed within 24 hours of life subsequently repeated every week until normal or discharge. Other routine therapies according to the management guideline of preterm infants. In general, routine clinical assistance and nursing will not be changed because of the study, out of the trial

intervention. No additional blood samples are required for this study.

Sample size Estimation

The sample size estimation was calculated by PASS software (2008 v8.0.3). According to our previous study, 28.7% (86/300) of preterm neonates with perinatal nARDS less than or equal to 34 weeks were diagnosed with BPD. An expected between-group difference was about 20% and an estimated 10% loss to follow-up, with 90% power and a 2-sided significance level of 0.05, 133 neonates would be needed at least in each group.

Statistical Analysis

Independent continuous variables, expressed as mean ± standard deviation, were analyzed using t test. Categorical variables were analyzed using $\chi2$ test or the Fisher's test. Quantitative variables would be firstly checked for normality using Kolmogorov-Smirnov test and were presented as mean ± standard deviation and compared using 2-tailed student's t test/one way-ANOVA, or median [interquartile range (IQR)] and compared with Mann-Whitney test. Qualitative variables were present as numbers and compared using the $\chi2$ test or the Fisher's test. We used SPSS 24.0 (SPSS, Chicago, IL, USA) for statistical analysis. For all analyses, a *P*-value < 0.05 was regarded as significant.

The following subgroup analysis will be performed: (1) Mild BPD or grade 1; moderate BPD or grade 2; severe BPD or grade 3; (2) mild ARDS: $4 \le OI < 8$； moderate ARDS：$8 \le OI < 16$； severe ARDS： $OI \ge 16$； (3) GA ≤ 28 weeks; 28 weeks < GA ≤ 32 weeks; 32 weeks < GA ≤ 34 weeks. (4) babies invasively ventilated ≤ 3 days or 3-7 days or > 7 days after birth. (5) HFOV only or HFOV plus VG.

Chongqing Health Centre for Women and Children

Chongqing Medical University

Study Title: Invasive Ventilation for Neonates With Acute Respiratory Distress Syndrome (ARDS)

PI: Long Chen, MD PhD, Senior Consultant, Chongqing Health Centre for Women and Children, Chongqing Medical University

Protocol version: 2.0

Protocol date: 1 Aug 2020

Protocol and statistical analysis plan

The use of elective high frequency oscillatory ventilation (HFOV) compared with conventional mechanical ventilation (CMV) has theoretical advantage in preterm infants with respiratory distress syndrome (RDS). Animal study also proves that, comparing with CMV, HFOV improves early lung function with sustained improvement in pulmonary mechanics out to 28 d, and has less pulmonary inflammation in the recovery phase of RDS. However, several clinical randomized controlled trials (RCTs) and meta-analyses comparing elective HFOV with CMV have shown inconsistent results. Meta-analyses indicate that heterogeneity is existed in study design, including baseline characteristics and outcomes measures.

Further study found that these RCTs above were all performed in the pre-neonatal acute respiratory distress syndrome (nARDS) era. We therefore speculate that a key heterogeneity may be the included criteria, and perinatal nARDS might be considered as RDS in the pre-nARDS era. Actually, these preterm infants in the two meta-analyses were diagnosed with acute pulmonary dysfunction, of which some were due to RDS, and the others were not detailed. A mixture of different ratio of RDS and nARDS in these studies may result in the inconsistent results. To date, there was no RCT comparing selective HFOV with CMV in preterm infants with perinatal nARDS.

The purpose of the present study was to explore whether selective HFOV was associated with less BPD as compared with CMV in preterm infants with perinatal nARDS.

Patients and Methods

Standard protocol approval, registration, and patient consent.

The study was approved by the Ethics Committee of Children's Hospital of Chongqing Medical University (project approval number:2018117) and registered at http://www.clinicaltrials.gov. (ID: NCT03591796) (the registration date: Nov-20, 2018). The registration name: Invasive Ventilation for Neonates With Acute

Respiratory Distress Syndrome(ARDS). It was from a prospective protocol, and informed parental written consents were obtained prior to study. The trial was performed in accordance with the approved guidelines and regulations of the participating institutions.

Study Design and Participants.

The RCT was conducted in two tertiary neonatal intensive care unit (NICU) from Aug, 2019 to December 2023, Children's Hospital of Chongqing Medical University, China and Chongqing Health Center for Women and Children, China. Eligibility requirements for neonates: (1) gestational age (GA) was less than or equal to 34 weeks; (2) preterm neonates were admitted to NICU , diagnosed with perinatal nARDS and stable supported by CMV; (3) Stabilization before randomization: $FiO_2 \leq 0.40$, Paw ≤10-14 cmH_2O, ≤ 40 bpm of respiratory rate, 90%-94% of SpO_2, pH > 7.20, $PaCO_2 \leq 60$ mmHg and > 35% of hematocrit (these may be evaluated by arterial blood gas analysis).

Neonates with at least one of the following criteria are not eligible for the study: (1) parents' decision not to participate; (2) major congenital anomalies or chromosomal abnormalities; (3) upper respiratory tract abnormalities; (4) need for surgery; (5) more than Grade 2 of intraventricular hemorrhage (IVH).

Allocation and Blinding

After obtaining parental consent, eligible preterm infants initially receiving CMV were randomly assigned to either HFOV or CMV group using a random number table and sealed, opaque envelopes. The envelopes were prepared and held by J.L., while allocation was overseen and executed by K.L.. Due to the nature of the intervention, we were unable to mask invasive supporting strategies from caregivers; however, parents or legal guardians and clinical investigators (Z.F. & Y.S.) were blinded to them. Additionally, statistical analysts (Q.Y., L.C. & L.-J.L.) remained blinded to the group allocations to maintain the integrity of the data analysis.

Study Intervention

Selective HFOV +/- volume guarantee (VG): Selective HFOV will only be provided with piston/membrane oscillators able to provide a real oscillatory pressure with active

expiratory phase (that is, Acutronic FABIAN-III, SLE 5000, Loweinstein Med LEONI+, Sensormedics 3100A). Other machines providing high frequency ventilations will not be included. The lung recruitment maneuver was performed as previously described, and lung volume was controlled by chest radiography/lung ultrasound to keep the right diaphragm at the level of 8-9 rib, 7-8 rib if airleak. The starting parameters were mean airway pressure of 8.0 cm of water (subsequent regulation range, 6-20, in step of 1 cm H_2O), frequency of 10 Hz (subsequent regulation range, 8-20, in step of 1 Hz), The ratio of inspiration to expiration fixed at 1:1, the amplitude of 20 (subsequent regulation range, 15-50, in steps of 5 cm H_2O), FiO_2 of 30% (subsequent regulation range, 21-100, in step of 5 cm H_2O) and high-frequency tidal volume (VThf) equaled 2.0 ml/kg if used (subsequent regulation range, 1.0-2.5, in step of 0.1 ml/kg/time). Subsequently, ventilator settings were adjusted at the discretion of the attending clinician to maintain a SpO_2 between 90%-95%, a PaO_2 between 50 and 80 mm Hg, a $PaCO_2$ between 35 and 60 mm Hg (PO_2 and PCO_2 levels were adjusted by transcutaneous monitors) and a PH between 7.20 and 7.45.

CMV: CMV was delivered by time-cycled, pressure-limited ventilators. Only pressure regulated volume control (PRVC) will be provided by any type of neonatal ventilator. The starting parameters were respiratory rate (RR) of 30 per minute (subsequent regulation range, 10-50, in steps of 2), inspiratory time(Ti) of 0.4 second (subsequent regulation range, 0.3-0.6, in steps of 0.05), peak inspiratory pressure (PIP) of 16 cmH_2O (subsequent regulation range, 6-30, in steps of 1), positive end-expiratory pressure (PEEP) of 6 cmH_2O (subsequent regulation range, 4-12, in step of 1) and FiO_2 of 30% (subsequent regulation range, 21-100, in step of 5 cm H_2O), tidal volume of 5ml/kg (subsequent regulation range, 3-6, in step of 0.5 ml/kg/time). Subsequently, ventilator settings were adjusted at the discretion of the attending clinician to maintain a SpO_2 between 90%-95%, a PaO_2 between 50 and 80 mm Hg and a $PaCO_2$ between 35 and 60 mm Hg (PO_2 and PCO_2 levels were adjusted by transcutaneous monitors) and a PH between 7.20 and 7.45.

The crossover to HFOV or CMV

The present study also covered a crossover design which supplied the included infants failing to respond to the assigned mode of ventilation with a trial of the alternate mode of ventilation. Analysis of results was done according to the neonate’s original group assignment.

Crossover criteria for neonates assigned to HFOV were failure for 3 hours to maintain an SpO2 ≥ 50 mmHg despite receiving a FiO_2 of 1.0, or failure for 3 hours to maintain a $PaCO_2$ ≤ 60 mmHg, or evidences of a ventilator-induced cardiac output decrease. Neonates who did not respond to CMV were returned to HFOV.

Crossover criteria for neonates assigned to CMV were failure for 3 hours to maintain an SpO_2 ≥ 50 mmHg despite receiving a FiO_2 of 1.0, or failure for 3 hours to maintain an PaCO2 ≤ 60 mmHg, or requiring > 30 cm H_2O of PIP to maintain adequate ventilation. Neonates who did not respond to HFOV were returned to CMV.

Weaning from invasive ventilation

Extubation requires fulfilling of all the following criteria: (1). Having received at least one loading dose of 20 mg/kg and 5-10 mg/kg daily maintenance dose of caffeine citrate; (2). pH > 7.20 $PaCO_2$ ≤55 mmHg (these may be evaluated by arterial blood gas analysis; (3). Paw ≤ 6 cmH_2O; (4). FiO_2 ≤ 0.30; (5). sufficient spontaneous breathing effort, as per clinical evaluation;(6).a hematocrit exceeding 35%. When a neonate had fulfilled the extubation criteria, gentle intratracheal and upper airways suction would then be done and nasal intermittent positive pressure ventilation would be started immediately.

Administration of Caffeine citrate, exogenous surfactant replacement, inhaled Nitric Oxide (iNO) and postnatal glucocorticoids.

Caffeine citrate: These preterm infants received caffeine citrate at a loading dose of 20 mg/kg and a maintenance dose of 5-10 mg/kg/d until 34 weeks of gestational age.

Exogeous surfactant replacement: No management guideline of nARDS was available. Therefore, the administration of surfactant was based on the European consensus guideline for the management of RDS. When the neonates were admitted to the NICU and had fulfilled the entry criteria, surfactant (Curosurf, Chiesi Pharmaceuticals, Parma, Italy; or Calsurf, Double-Crane Pharmaceutical Co., Ltd,

Beijing, China) was administered according to the manufacture's instruction as a rescue treatment if an infant needed $FiO_2 > 0.30$ or invasive ventilation to maintain the targeted SpO_2 with 90%-94%. The intervals of surfactant administration were 6 to 12 hours.

iNO: iNO was enforced if SpO_2 was less than 80% after $FiO_2 > 60\%$ or diagnosed with persistent pulmonary hypertension of newborn (PPHN). These preterm infants received iNO at a loading dose of 20ppm for 24 h and a maintenance dose of 5-10 ppm for the next six days.

Eligibility criteria for re-intubation.

The subjects would be re-intubated if they were not improved and needed invasive ventilation. The criteria for re-intubation and mechanical ventilation were one of the following conditions reached: 1. Cardio-respiratory arrest or any types of pulmonary hemorrhage; 2. Two hours of respiratory acidosis with $PaCO_2 > 70$ mmHg and pH < 7.2; 3. Two hours of hypoxia with $PO_2 < 50$ mmHg, under 0.6 of FiO_2 and maximal pressures given of 16 cm H_2O; 4. Apnea 3 times per hour and heart rate less than 100/min; 5. Requiring mask ventilation in any case; 6. Persistent low blood pressure without responsive to liquid resuscitation and vasoactive agents. If a preterm infants failed to the first extubation, the re-needed invasive modes and noninvasive supporting mode of the second extubation was at the discretion of the attending neonatologist.

Termination of the study

The study would end if one of the following conditions was reached: 1. Death; 2. Parents' decision not to continue the participation; 3. Discharge according to doctors' suggestions.

The primary and secondary outcomes.

The primary outcomes will be: (1) the incidence of BPD, according to the NICHD definition in 2001.

The secondary outcomes were to assess the outcomes between the two groups, including the incidences of BPD or death, death, air leak (pneumothorax and/or pneumomediastinum), haemodynamically significant patent ductusarteriosus (hsPDA), retinopathy of prematurity (ROP) > 2nd stages, necrotizing enterocolitis (NEC) ≥ 2nd

stages, and IVH > 2nd grades.

Monitoring and treatments

All neonates will be continuously monitored for SpO_2, ECG, heart and respiratory rate. $PaCO_2$ will be monitored using arterial blood gas analysis and/or transcutaneous monitors according to the Chinese Association of Pediatrics guidelines and the manufacturer's recommendations. Frequency of blood gas analysis will be decided by the attending clinicians. Furthermore, the following treatments or tests will be provided: 1). Placement of umbilical central venous catheter and/or peripherally inserted central venous lines. 2). Ultrasound was used to assess cardiac morphology, pulmonary pressures and PDA within the first 24 hours of life and subsequently repeated every week until normal/leaving hospital. 3) Cerebral ultrasound was assessed within 24 hours of life subsequently repeated every week until normal or discharge. Other routine therapies according to the management guideline of preterm infants. In general, routine clinical assistance and nursing will not be changed because of the study, out of the trial intervention. No additional blood samples are required for this study.

Sample size Estimation

No previous RCT were performed comparing HFOV vursus CMV in preterm infants with NARDS. According to prior RCTs comparing HFOV with CMV in preterm infants, reported differences in outcomes ranged from 4.5% to 37.5% among infants with respiratory insufficiency at 26–30 weeks' gestation. To ensure a conservative estimate for our study population, we adopted a median effect size of 13% in BPD incidence between HFOV and CMV treatment. Based on an assumed baseline incidence of 28.7% among preterm neonates born ≤34 weeks and diagnosed with NARDS in our population, we estimated that a minimum of 320 neonates (160 per group) would be required to achieve 80% power at a two-sided significance level of 0.05. Sample size calculation was performed using PASS software (version 8.0.3, 2008).

Statistical Analysis

Independent continuous variables, expressed as mean ± standard deviation, were analyzed using t test. Categorical variables were analyzed using $\chi 2$ test or the Fisher's

test. Quantitative variables would be firstly checked for normality using Kolmogorov-Smirnov test and were presented as mean ± standard deviation and compared using 2-tailed student's t test/one way-ANOVA, or median [interquartile range (IQR)] and compared with Mann-Whitney test. Qualitative variables were present as numbers and compared using the χ2 test or the Fisher's test. We used SPSS 24.0 (SPSS, Chicago, IL, USA) for statistical analysis. For all analyses, a *P*-value < 0.05 was regarded as significant.

The following subgroup analysis will be performed: (1) Mild BPD; moderate BPD; severe BPD; (2) mild ARDS: 4 ≤ OI < 8；moderate ARDS：8 ≤ OI < 16；severe ARDS：OI ≥ 16； (3) GA ≤ 28 weeks; 28 weeks < GA ≤ 32 weeks; 32 weeks < GA ≤ 34 weeks. (4) babies invasively ventilated ≤ 3 days or 3-7 days or > 7 days after birth. (5) HFOV only or HFOV plus VG.
