# Supplementary material for: High-Frequency Oscillation vs Mechanical Ventilation for Neonatal Acute Respiratory Distress Syndrome: A Randomized Clinical Trial
Source: JAMA Netw Open. 2026 Mar 9;9(3):e260268. doi: 10.1001/jamanetworkopen.2026.0268 (PMC12973098; doi:10.1001/jamanetworkopen.2026.0268)
Supplement: Supplement 3. — Data Sharing Statement [file jamanetwopen-e260268-s003.pdf]

Data Sharing Statement

Li. High-Frequency Oscillation vs Mechanical Ventilation for Neonatal Acute Respiratory Distress Syndrome. *JAMA Netw Open*. Published March 09, 2026.
doi:10.1001/jamanetworkopen.2026.0268

Data
Additional Information: http://www.clinicaltrials.gov (NCT03591796) (the registration date: Nov-20, 2018, Invasive Ventilation for Neonates With Acute Respiratory Distress Syndrome(ARDS))
Data available: Yes
Data types: Other (please specify)
Additional Information: the data underlying the findings in this paper are openly and publicly available and can be found here: https://data.mendeley.com/v1/datasets/publish-confirmation/9jdr9njps2/1.
How to access data: the data underlying the findings in this paper are openly and publicly available and can be found here: https://data.mendeley.com/v1/datasets/publish-confirmation/9jdr9njps2/1.
When available: With publication

Supporting Documents
Document types: Statistical/analytic code
How to access documents: the data underlying the findings in this paper are openly and publicly available and can be found here: https://data.mendeley.com/v1/datasets/publish-confirmation/9jdr9njps2/1.
When available: With publication

Additional Information
Who can access the data: corresponding author
Types of analyses: non-profit
Mechanisms of data availability: investigator support
